# SPNing our wheels—Pancreatic solid pseudopapillary neoplasm as an extraluminal etiology of persistent duodenal ulceration

**DOI:** 10.1002/jpr3.70003

**Published:** 2025-02-25

**Authors:** Kanak V. Kennedy, Jibraan A. Fawad, Y. Dana Neugut, Doris Valenzuela‐Araujo, Alexander Coe, Tricia R. Bhatti, Michael Acord, Michael D. Manfredi, Petar Mamula, Kathleen M. Loomes, Jefferson N. Brownell

**Affiliations:** ^1^ Division of Gastroenterology, Hepatology, and Nutrition, Department of Pediatrics Children's Hospital of Philadelphia Philadelphia Pennsylvania USA; ^2^ Division of Pediatric Gastroenterology, Hepatology, and Nutrition, Department of Pediatrics Stanford University School of Medicine Palo Alto California USA; ^3^ Department of Pathology and Laboratory Medicine Children's Hospital of Philadelphia Philadelphia Pennsylvania USA; ^4^ Department of Radiology Children's Hospital of Philadelphia Philadelphia Pennsylvania USA; ^5^ Perelman School of Medicine University of Pennsylvania Philadelphia Pennsylvania USA

**Keywords:** duodenal ulcer, pancreas, solid pseudopapillary mass, upper GI bleeding

## Abstract

Pediatric upper gastrointestinal (GI) bleeding secondary to duodenal ulceration is a potentially serious and life‐threatening condition with a broad differential diagnosis. We present a pediatric case of a pancreatic head solid pseudopapillary neoplasm (SPN) presenting with duodenal ulceration and recurrent upper GI bleeding. This case highlights pancreatic SPNs as a rare extrinsic cause of duodenal ulceration. Recurrence and progression in size and extent of a duodenal ulceration in the absence of other inciting factors should raise suspicion for an extraluminal etiology.

## 1 INTRODUCTION

Pediatric upper gastrointestinal (GI) bleeding is a potentially serious and life‐threatening condition, with mortality rates as high as 5%–21% depending on the underlying etiology.^1^ Effective identification and management of the underlying condition is crucial to reducing morbidity and mortality. Here, we present a case of an adolescent female who presented with recurrent upper GI bleeding secondary to persistent duodenal ulceration.

## 2 CASE REPORT

A 14‐year‐old female presented to our tertiary care center emergency department with a 5‐day history of progressive fatigue and dizziness, and was found to have significant normocytic anemia (hemoglobin 5.8 g/dL, normal 12.0–16.0 g/dL; hematocrit 19.8%, normal 36.0%–46.0%; mean corpuscular volume 78 fL, normal 78.0–102.0 fL). She denied any associated syncopal episodes, focal weakness, or vision changes, but on further questioning reported intermittent melenic stools. She denied upper GI tract symptoms, including nausea, vomiting, dysphagia, abdominal pain, or hematemesis. She had no associated weight loss. Her past medical history was notable for sacrococcygeal teratoma in infancy, diagnosed in utero and treated with chemotherapy and surgical resection. She had no family history of inflammatory GI disease or bleeding/clotting disorders. She denied any prior nonsteroidal anti‐inflammatory drug (NSAID) use and was not on any scheduled or as needed medications before admission.

**Figure 1:**
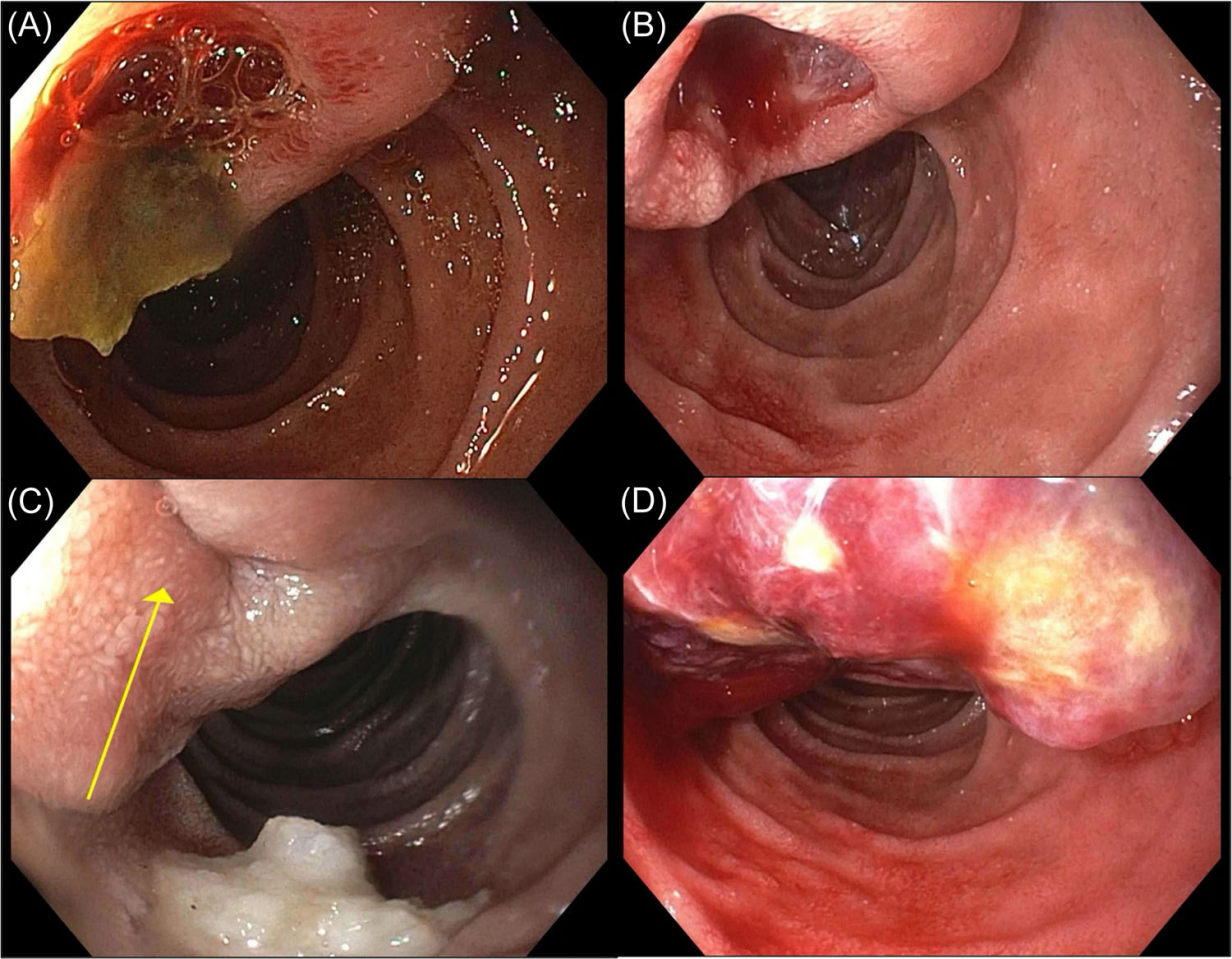
Endoscopic evolution of patient's duodenal ulceration appearance. Duodenal ulcer images at time of (A) initial presentation, (B) subsequent episode of recurrent anemia and abdominal pain, (C) surveillance endoscopy during asymptomatic period, and (D) at time of SPN diagnosis. SPN, solid pseudopapillary neoplasm.

Further evaluation revealed positive fecal occult blood, significant iron deficiency (ferritin 2.1 ng/mL, normal 10.0–67.4 ng/mL; transferrin 377 mg/dL, normal 180–370 mg/dL; transferrin saturation 2.0%, normal 6%–40%), normal prothrombin time, partial thromboplastin time, and international normalized ratio, negative *Helicobacter pylori* stool antigen, normal fecal calprotectin, and negative infectious stool studies. Bowel ultrasound showed no bowel wall thickening or hyperemia and a magnetic resonance (MR) abdomen/pelvis without intravenous contrast showed no tumor recurrence. Esophagogastroduodenoscopy (EGD) was performed and demonstrated a 10‐mm Forrest IIb cratered ulcer with an adherent clot in the second portion of the duodenum adjacent to the major papilla (Figure 1A). Biopsies obtained adjacent to the ulcer demonstrated no pathologic diagnosis, including no evidence of *H. pylori* infection. Colonoscopy was visually and histologically normal. She was started on high‐dose acid suppression with omeprazole (40 mg twice daily) and discharged with plan for close outpatient follow‐up.

Given symptomatic improvement without recurrence of melena, omeprazole was weaned after 4 months to 40 mg once daily. After 3 months, the patient's hemoglobin decreased from 12.0 to 9.5 g/dL (normal 12.0–16.0 g/dL). This prompted repeat endoscopic evaluation, which demonstrated a larger 20 mm Forrest Ib cratered duodenal ulcer with active bleeding (Figure 1B). Using a duodenoscope, the ulcer was treated with epinephrine and argon plasma coagulation (APC). She resumed high‐dose omeprazole (40 mg twice daily) following endoscopy. Additional evaluation at this time was notable for normal serum gastrin while on omeprazole therapy (83 pg/mL, normal 0–100 pg/mL). Surveillance EGD performed 6 months later demonstrated a healing ulcer with intact overlying mucosa (Figure 1C). Hemoglobin was stable at 13.0 g/dL (normal 12.0–16.0 g/dL) at the time of endoscopy.

**Figure 2:**
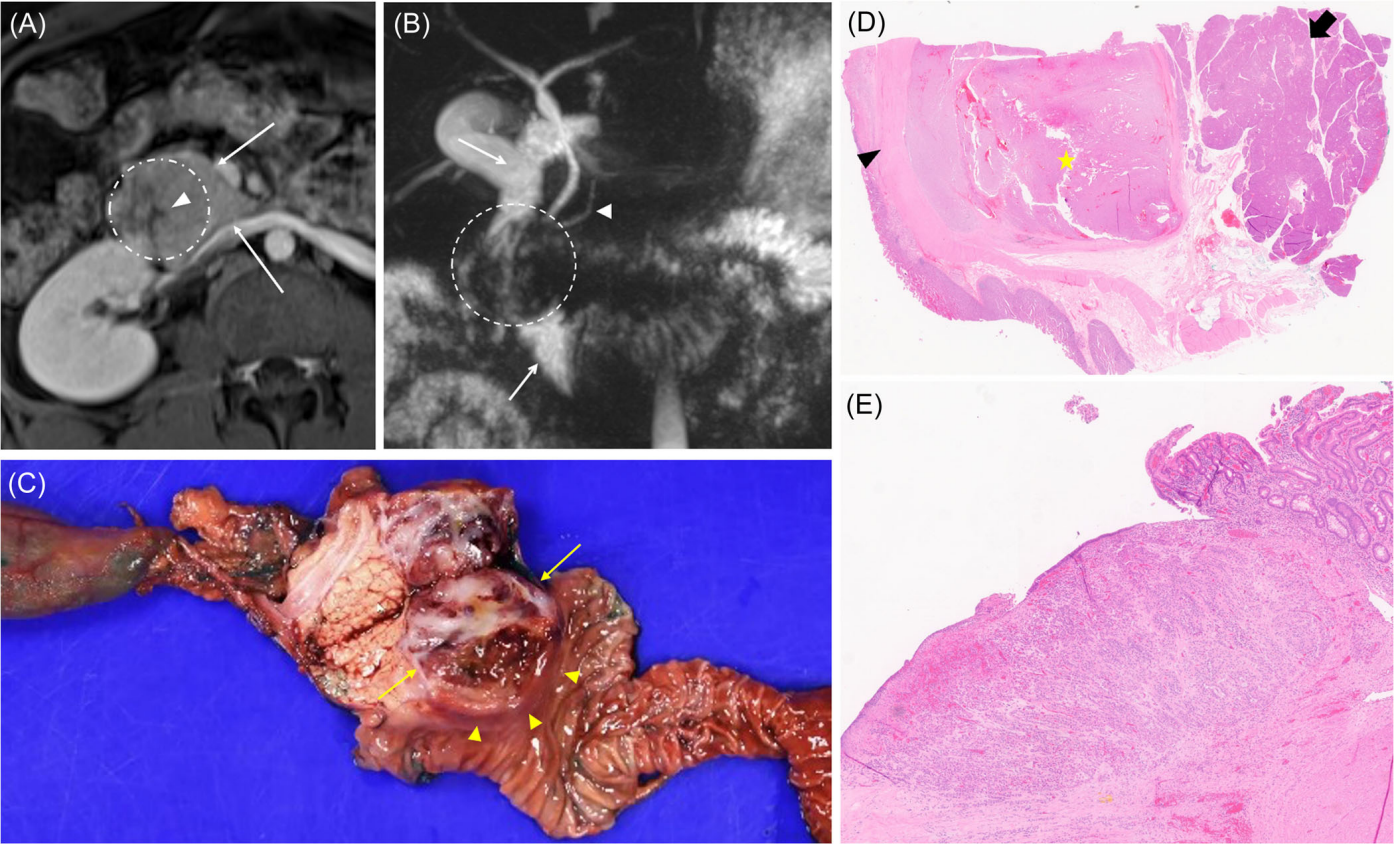
Radiographic imaging and histopathology confirming pancreatic solid pseudopapillary neoplasm. (A) Axial T1 fat‐saturated post‐contrast MR image demonstrates an enhancing mass (dashed circle) within the head of the pancreas (thin arrows). Central ulceration is shown as an area of hypoattenuation (arrowhead). (B) Coronal T2 SPACE MRCP shows a hypointense mass (dashed circle) protruding into the lumen of the duodenum (thin arrows). There was no biliary or pancreatic (arrowhead) duct dilation. (C) Gross images of resected tumor (thin arrows) and adjacent bowel (arrowheads) post‐pylorus‐preserving pancreaticoduodenectomy. (D) Histology of resected tumor (star) located between muscularis propria of the duodenum (black arrowhead) and adjacent, nonneoplastic pancreas (black arrow). Tumor consists of a partially encapsulated proliferation of cytologically bland tumor cells with areas of discohesion and pseudopapillae formation (hematoxylin and eosin stain, 15× original magnification). (E) Higher power view of ulcerated duodenal mucosa overlying tumor (hematoxylin and eosin stain, 200× original magnification). MR, magnetic resonance; MRCP, magnetic resonance cholangiopancreatography.

The patient presented again 4 months later with 2 weeks of worsening epigastric pain, presyncope, and recurrent melena. Admission laboratory studies demonstrated recurrent severe anemia (Hgb 6.5 g/dL, normal 12.0–16.0 g/dL). She underwent urgent endoscopy, which now showed a new ulcerated mass 25 mm in diameter, protruding into the lumen and distorting the papilla at the previously noted ulcer site (Figure 1D). After epinephrine injection, the bleeding vessel was desiccated with APC, and the mass was biopsied with standard forceps. An MR cholangiopancreatography (MRCP) was obtained while awaiting histopathology analysis, which demonstrated a heterogeneous enhancing lesion in the pancreatic head measuring 4.8 × 4.0 × 3.6 cm^3^ (Figure 2A,B). Biopsy results subsequently confirmed a diagnosis of a pancreatic solid pseudopapillary neoplasm (SPN).

Primary resection of the tumor with a pylorus‐preserving pancreaticoduodenectomy was performed without complication (Figure 2C). Eight months after resection, the patient has had no further episodes of upper GI bleeding or symptomatic anemia, and surveillance imaging has shown no signs of recurrence.

## 3 DISCUSSION

We report a rare presentation of pancreatic head SPN in a pediatric patient presenting with duodenal ulceration and upper GI bleeding. Pancreatic SPNs represent only 2%–3% of all primary pancreatic tumors, most frequently affecting young females.^2^, ^3^ They typically occur in the pancreatic tail, although pancreatic head lesions can occur more often in adolescents. Clinical symptoms associated with SPNs are often nonspecific, including abdominal pain, nausea, bloating, early satiety, and vomiting. They are slow‐growing and have relatively low malignant potential, with a reported 5‐year survival rate of 98%.^2^, ^4^ Up to 10%–15% of cases demonstrate locally aggressive behavior,^3^, ^4^, ^5^, ^6^ such as invasion of adjacent structures as was seen in our patient. Risk factors for invasive tumor behavior of malignant SPNs include larger tumor size, increased Ki‐67 index, and lymphovascular invasion.^3^

The differential diagnosis for persistent duodenal ulcers includes drug‐induced injury secondary to NSAIDs and corticosteroid use, *H. pylori* infection, acid hypersecretory states including Zollinger–Ellison syndrome, inflammatory diseases (Crohn's disease and eosinophilic GI disease), vascular insufficiency, malignancy, foreign bodies, obstruction, and postoperative anastomotic ulceration. In this case, endoscopic biopsies of the mucosa surrounding the patient's ulcer site were obtained on several occasions, with histopathology reassuring against underlying infection and inflammatory diseases. Further, she had no history of NSAID or corticosteroid exposure, and serum gastrin levels were within normal limits on acid suppression.

**Figure 3:**
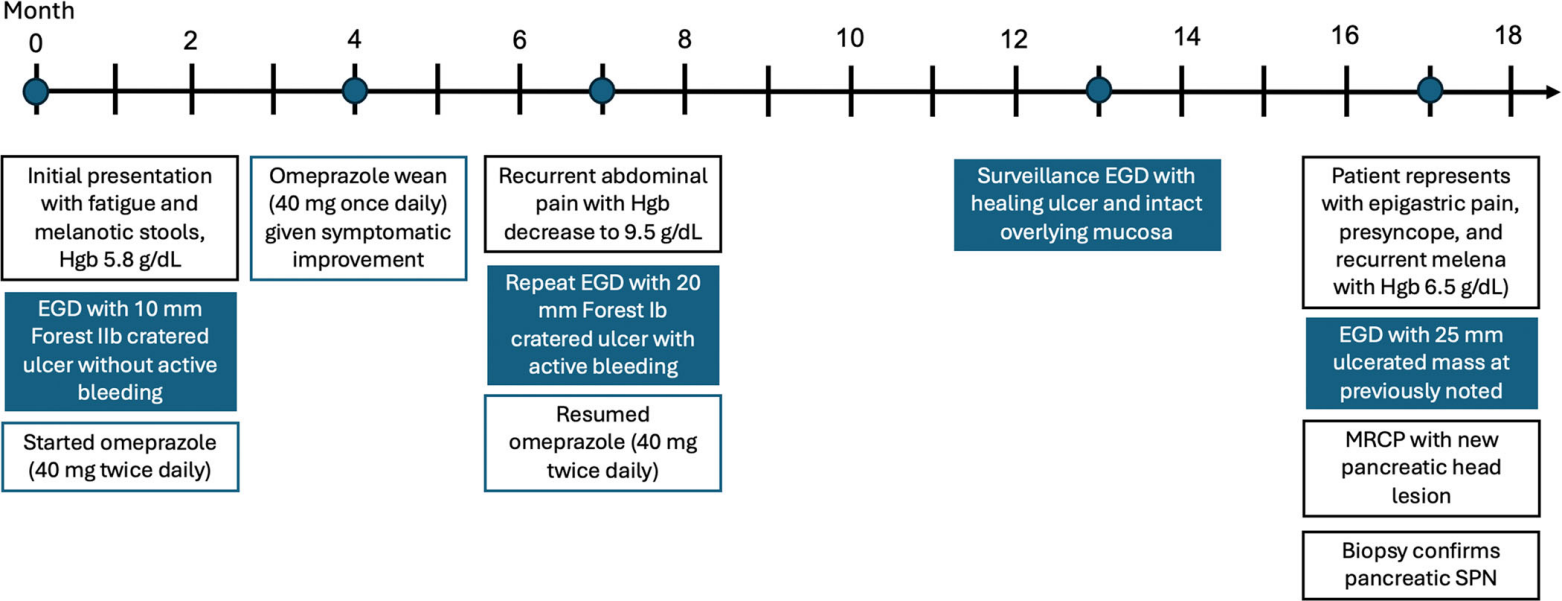
Timeline of patient's clinical course from initial presentation to SPN diagnosis. EGD, esophagogastroduodenoscopy; Hgb, hemoglobin; mg, milligram; MRCP, magnetic resonance cholangiopancreatography; SPN, solid pseudopapillary neoplasm.

Of note, our patient was evaluated with cross‐sectional imaging with MR abdomen/pelvis without intravenous contrast at the time of her initial presentation, months before her definitive diagnosis (Figure 3). While the imaging was tailored to evaluate for recurrent germ cell tumors given the patient's past medical history, retrospective comparison of that study to her subsequent MRCP at diagnosis showed a small enhancing mass in the head of the pancreas measuring 3 × 2.2 × 2 cm^3^ (anteroposterior × transverse × craniocaudal) that was not appreciated at the time of the original study. The evolving imaging findings during our patient's 2‐year clinical course are consistent with the natural history of locally aggressive pancreatic SPNs described in the literature.^7^

This case highlights pancreatic SPNs as a rare extrinsic cause of duodenal ulceration. Recurrence and progression in size and extent of a duodenal ulceration in the absence of other inciting factors should raise suspicion for an extraluminal etiology.

## CONFLICT OF INTEREST STATEMENT

The authors declare no conflicts of interest.
